# Extracellular vesicles from hydroxycamptothecin primed umbilical cord stem cells enhance anti-adhesion potential for treatment of tendon injury

**DOI:** 10.1186/s13287-020-02016-8

**Published:** 2020-11-25

**Authors:** Juehong Li, Zhixiao Yao, Hao Xiong, Haomin Cui, Xu Wang, Wei Zheng, Yun Qian, Cunyi Fan

**Affiliations:** 1 Department of Orthopaedic Surgery, Shanghai Jiao Tong University Affiliated Sixth People’s Hospital, No. 600 Yishan Road, Shanghai, China; 2 Youth Science and Technology Innovation Studio of Shanghai Jiao Tong University School of Medicine, Shanghai, 200025 China

**Keywords:** Extracellular vesicles, Human umbilical cord stem cells, Hydroxycamptothecin, Tendon adhesion

## Abstract

**Background:**

Peritendinous fibrosis represents a fibrotic healing process that usually occurs after tendon injury or surgery. This worldwide challenge hampers the functional rehabilitation and the mobility of extremities. However, effective treatment is still lacking at present. The aim of our study was to explore the effect of extracellular vesicles derived from hydroxycamptothecin primed human umbilical cord stem cells (HCPT-EVs) on post-traumatic tendon adhesion.

**Methods:**

Extracellular vesicles derived from unprimed human umbilical cord mesenchymal stem cells (Unprimed EVs) or HCPT-EVs were isolated and characterized. A rat model of Achilles tendon injury was used to confirm the anti-adhesion effect of HCPT-EVs and compared with that of Unprimed EVs in vivo. In vitro, the inhibitory effects of HCPT-EVs on fibroblast proliferation, viability, and myofibroblast differentiation upon TGF-β1 stimulation were compared with the effects of Unprimed EVs. For mechanistic analysis, the expression of endoplasmic reticulum stress (ERS)-associated proteins was examined among the effector cargos of HCPT-EVs and Unprimed EVs. The ERS antagonist salubrinal was used to determine the ERS dependence of the anti-adhesion effects of HCPT-EVs.

**Results:**

There were no obvious differences between Unprimed EVs and HCPT-EVs in terms of morphology, particle size, characteristic protein expression, and cellular uptake. HCPT-EVs exhibited a fortified anti-adhesion effect after Achilles tendon injury compared with Unprimed EVs. Fibroblast proliferation and viability and myofibroblast differentiation were all inhibited by HCPT-EVs. These properties were superior for HCPT-EVs relative to Unprimed EVs. Mechanistically, HCPT-EVs contained more ERS-associated protein than Unprimed EVs and activated the ERS pathway in fibroblast to counteract myofibroblast differentiation.

**Conclusion:**

This study demonstrates that HCPT-EVs show high anti-adhesion potential for the treatment of tendon injury by provoking ERS in fibroblasts. HCPT-EVs represent a promising strategy for clinical use in treating adhesion-related diseases.

## Backgroud

Tendons are one of the most vulnerable sites to trauma but has poor endogenous healing ability [1]. Tendon injury can, even after careful surgical repair, lead to fibrotic tissue ingrowth followed by tendon fibrosis [2]. The consequent adhesion impairs the natural tendon structure, preventing tendon gliding and finally causing extremity stiffness. According to published statistics from the USA, injuries in the hand and wrist or Achilles tendon can occur at an incidence rate of 2.66 injuries per 1000 person-years and 33.2 injuries per 100,000 person-years, respectively [3, 4], and the resulting tendinopathy adds tens of billions of dollars per year to medical expenses [5]. Thus, the development of an effective anti-adhesion therapy is imperative for both raising patients’ quality of life and reducing socioeconomic burden.

In our previous study, we demonstrated that hydroxycamptothecin (HCPT), a natural camptothecin compound found in *Camptotheca acuminata*, acts as a DNA topoisomerase I inhibitor to suppress cell overproliferation and induce apoptosis of fibroblasts, which remarkably prevents tendon adhesion after tendon injury [6]. However, the DNA synthesis inhibitory effect of HCPT could also lead to concerns about its toxicity to normally functioning mammalian cells [7]. Accordingly, direct clinical application of HCPT could give rise to severe symptoms such as myelosuppression, nausea, and diarrhea [8]. Moreover, its aqueous solubility, stability in blood circulation, and concentration fluctuations should be taken into consideration when directly translating HPCT into clinical application. To take advantage of HCPT in tendon adhesion prevention but avoid its potential safety issues, a novel strategy for HCPT usage is warranted.

Recent years have witnessed the emergence of extracellular vesicles (EVs) as a desirable therapeutic modality. Satisfactory results were obtained for inflammation resolution and tendon healing by EVs derived from bone marrow mesenchymal stem cells (BMSCs), which showed good prospects for EVs application after tendon injury [9, 10]. These natural nanosized (30–150 nm in diameter) membrane-bound particles have the advantages of high biosafety and good in vivo stability and are thus superior vehicles for effector molecule delivery [11]. As important communicators involved in intercellular interactions, EVs carry biomolecules such as miRNAs and proteins with the ability to modulate relevant signaling events and therefore shape the biological response to diverse stimuli [12]. It has also been established that cargos of EVs can be launched or modified to achieve enhanced function. For example, miR-199a-modified EVs isolated from lentivirus-transfected adipose tissue-derived mesenchymal stem cells (ADSCs) fostered chemosensitivity of hepatocellular carcinoma via mTOR pathway inhibition [13], and mmu_circ_0001359-enriched EVs generated through mmu_circ_0001359 overexpression in ADSCs more effectively ameliorated airway fibrosis by shifting macrophage polarization [14]. Furthermore, specific conditional pretreatment could even endow EVs with cargos exerting integrated effects and synergistically facilitate their particular therapeutic application [15, 16]. Thus, it is reasonable to infer that HCPT pretreatment of producer cells could also bring forth reliable modified EVs, which may be a promising candidate to treat adhesion after tendon injury and circumvent HCPT direct application.

Human umbilical cord mesenchymal stem cells (HUMSCs) are now one of the most popular cell sources of EVs due to their ease of accessibility, high productivity and purity, and lack of ethical issues. A growing number of clinical trials have also implicated HUMSCs in cell-based therapy for numerous disorders ranging from tissue damage to homeostatic imbalance [17–19]. Surprisingly, EVs from human umbilical cord stem cells (Unprimed EVs) themselves have the intrinsic ability to diminish adhesion and fibrotic responses, which have already been observed in acute kidney injury and scar formation [20, 21]. To further enhance its anti-adhesion property, we pretreated HUMSCs with HCPT and hypothesized that EVs from HCPT-primed HUMSCs (HCPT-EVs) could exert a stronger treatment effect on tendon adhesion after tendon injury.

In this study, the beneficial effect of HCPT-EVs on tendon adhesion was assessed in a rat model of tendon injury. The inhibitory effect of HCPT-EVs on cell proliferation and myofibroblast differentiation upon TGF-β1 stimulation in fibroblasts was also evaluated in vitro. In addition, we explored endoplasmic reticulum stress (ERS) signaling to determine whether the enhanced anti-adhesion capability of HCPT-EVs was related to the increased ERS cargos induced by HCPT pretreatment.

## Materials and methods

### Materials

HCPT was purchased from Santa Cruz (CA, USA) and dissolved in dilute alkali solution followed by further dilution with normal saline or culture medium. TGF-β1 was purchased from Minneapolis (MN, USA). Fetal bovine serum (FBS) was obtained from Gibco (Carlsbad, CA, USA). High glucose Dulbecco’s modified Eagle’s medium (DMEM), alpha minimum essential medium (α-MEM), and penicillin/streptomycin were purchased from HyClone (Logan, UT, USA). Anti-CD9, anti-63, anti-ALIX, anti-TSG101, anti-calnexin, anti-CHOP, anti-Bcl-2, anti-α-smooth muscle actin (α-SMA), and anti-COL III antibodies were provided by Abcam Biotechnology (Cambridge, MA, USA); anti-Bax, anti-glucose regulated protein 78 (GRP78), and anti-β-actin antibodies were purchased from Cell Signaling Technology (CA, USA).

### Isolation and characterization of EVs

For EVs isolation, EV-depleted FBS was prepared by centrifuging FBS at 100,000*g* for 18 h, and only the upper four-fifths of the supernatant was collected. EV-depleted FBS at 10% was added to basic α-MEM culture medium to make EV-depleted complete culture medium. When the confluence of HUMSCs reached 50%, the culture medium was changed to the EV-depleted complete culture medium, and cells were further cultured for 48 h. Supernatants were collected for EVs’ isolation. For the HCPT priming strategy, HUMSCs were initially incubated in 10% FBS-supplemented α-MEM in the presence of 1 μg/ml HCPT for 24 h, and then, the medium was refreshed with EV-depleted complete culture medium and further incubated for 48 h. Finally, supernatants were collected for subsequent EVs isolation.

The isolation of EVs was performed as previously described with minor modifications [22]. Briefly, the collected supernatants were first centrifuged at 300*g* for 10 min followed by 2000*g* for 20 min at 4 °C to remove cells. Cell debris was then removed by further centrifuging the above supernatants at 10,000*g* for 30 min. After that, supernatants were harvested and ultracentrifuged at 120,000*g* for 70 min at 4 °C. Precipitated pellets were then resuspended using phosphate-buffered saline (PBS) and were again ultracentrifuged at 120,000*g* for 70 min at 4 °C. EVs were ultimately obtained by discarding the supernatants and resuspending the precipitates.

Transmission electron microscopy (TEM) was used to observe and identify the morphology of the EVs. The size distribution and particle concentration were determined by nanoparticle tracking analysis (NTA; ZetaView PMX 120, Particle Metrix, Meerbusch, Germany). The expression of EV markers was examined using western blotting (WB).

### Cell lines and culture

The rat fibroblast cell line and HUMSCs were both obtained from the Cell Bank of the Chinese Academy of Sciences. HUMSCs were isolated from the umbilical cord of one healthy donor and passaged for expansion, while the rat fibroblast cell line was initially isolated from the skin tissue of one healthy donor and subjected to immortalization. The fibroblast cell line was cultured in high glucose DMEM supplemented with 10% FBS and 1% penicillin/streptomycin. HUMSCs were maintained in α-MEM supplemented with 10% FBS and 1% penicillin/streptomycin. HUMSCs at passages 4–6 were used in this study. A constant temperature setting at 37 °C and an atmosphere containing 5% CO2 were applied to maintain cell culture.

For subsequent fibroblast cell experiments, 2 ng/ml TGF-β1 and 100 μg/mL Unprimed EVs or HCPT-EVs were applied simultaneously. RNA was extracted at 24 h, and proteins were obtained at 48 h unless mentioned specifically.

### Cell proliferation and viability

Cell proliferation at days 1 to 4 was tested by CCK8 analysis. Fibroblasts were inoculated into 96-well plates and cultured with or without TGF-β1 in combination with Unprimed EVs or HCPT-EVs. After the indicated time points, 10 μl of CCK8 solution (Dojindo, Kumamoto, Japan) was added to each well, and the cells were further incubated for 3 h. Cell proliferation was then acquired and compared by recording the absorbance at 450 nm with a microplate reader (Multiskan MK3; ThermoFisher Scientific, America).

Live/dead staining was performed to check the effect of Unprimed EVs or HCPT-EVs on cell viability in the presence of TGF-β1 using a live/dead staining kit according to the manufacturer’s instructions. Briefly, after 48 h of incubation, cells were rinsed twice with Dulbecco’s phosphate-buffered saline (D-PBS) and incubated with the staining solution containing calcein AM and ethidium homodimer-1 for 30 min at ambient temperature. Fluorescence was observed under microscopy. Five parallel replicates were performed for dead/live rate quantification.

### Immunofluorescence staining

Immunofluorescence staining was performed as previously described [23]. Briefly, after fixation in 4% paraformaldehyde for 20 min, cells were permeabilized with 0.5% Triton X-100. Then, the cells were blocked using 1% BSA and incubated with anti-COL III (dilution: 1:100) and anti-α-SMA (dilution: 1:100) antibodies overnight at 4 °C. The next day, the cells were washed with PBS three times and further incubated with Alexa Fluor 488-conjugated or Cy3-conjugated secondary antibodies (Servicebio, Wuhan, China, dilution: 1:200) in the dark for 1 h at room temperature. 4,6-Diamidino-2-phenylindole (DAPI) was applied to counterstain the nuclei. Photos were taken by a digital slide scanner (Pannoramic MIDI; 3DHISTECH Ltd).

### PKH67 staining

To visualize the uptake of EVs in fibroblasts, EVs were labeled with PKH67 according to the manufacturer’s protocol. Briefly, the isolated EVs were incubated with PKH67 dye solution (Sigma, MO, USA) for 5 min. Then, the mixed EV/PKH67 solution was quenched with EV-depleted FBS, filtered through a 0.22-μm membrane filter and centrifuged at 12,000*g* for 70 min to remove the excess dye. The obtained labeled EVs were washed with PBS 2 times and then cocultured with HUMSCs for 24 h. After staining the nucleus with DAPI (4′,6-diamidino-2-phenylindole; Beyotime, Shanghai, China) according to the manufacturer’s instructions, images were taken using a fluorescence microscope (Eclipse TS100; Nikon Corporation, Tokyo, Japan).

### Rat Achilles tendon injury model

Thirty-three adult male Sprague-Dawley (SD) rats weighing 250 to 300 g were purchased from Shanghai SLAC Laboratory Animal Co., Ltd. (Shanghai, China) and were housed under specific pathogen-free (SPF) conditions. After accommodating the environment for 1 week, rats were subjected to Achilles tendon injury to establish the fibrotic healing response. Anesthesia was performed using pentobarbital sodium (40 mg/kg). Hair on each left hind limb was carefully removed by a razor, and skin was sterilized by iodophor swab. The Achilles tendon was exposed by an “S”-shaped incision. The thin strand of the Achilles tendon was resected, and the thick strand of the Achilles tendon was transected in the middle. The cut at the thick strand of the Achilles tendon was repaired using the 6-0 polypropylene suture (Ethicon, Edinburgh, UK). Then, rats were randomly distributed into 3 groups (eleven animals for each treatment group): the adhesion group (tendon injury and vehicle injection), Unprimed EV group (tendon injury and Unprimed EV injection), and HCPT-EV group (tendon injury and HCPT-EV injection). Unprimed EVs and HCPT-EVs were both subcutaneously injected at the injury site at a dose of 200 μg after skin closure, while rats in the adhesion group were subcutaneously injected with the same volume of PBS (50 μl).

All of the above-described experimental protocols were approved by the Institutional Animal Care and Use Committee (IACUC) of the Shanghai Sixth People’s Hospital (DWLL2020-0556), and all experimental procedures were performed in strict keeping with the policy announced by the IACUC of Shanghai Sixth People’s Hospital and the Animal Management Regulations of China (1988 and revised in 2001, Ministry of Science and Technology).

### Histopathological observation and immunohistochemical staining

Three weeks after surgery, rats were sacrificed, and the entire hindlimb was harvested and fixed in 10% (v/v) formalin. Then, the well-fixed tissues were dehydrated by standard procedures and embedded in paraffin. Sections were made longitudinally with a thickness of approximately 6 μm and mounted onto slides. To isolate protein for WB analysis, harvested Achilles tendons were snap-frozen in liquid nitrogen and stored at − 80 °C.

The severity of adhesion was semiquantified according to a previously described adhesion grading scoring system through macroscopic observation [24]. The scoring system consists of 5 levels: (1) macroscopically no presence of adhesion tissue, (2) presence of adhesion tissue required to be separated by blunt dissection, (3) presence of less than 50% of adhesion tissue required to be separated by clear and sharp dissection, (4) presence of 51~97.5% of adhesion tissue required to be separated by clear and sharp dissection, and (5) presence of more than 97.5% of adhesion tissue required to be separated by clear and sharp dissection. A previously reported histological adhesion scoring system was used to further evaluate the adhesion degree histologically [21], including scores of 4 grades: (1) histologically no presence of adhesion tissue, (2) histologically presence of adhesion area less than 33% of tendon surface, (3) histologically presence of adhesion area between 33 and 66% of tendon surface, and (4) histologically presence of adhesion area more than 66% of tendon surface. A histological healing scoring system was also applied to assess the tendon healing outcomes histologically, rating by 4 grades: (1) histologically presence of good continuity and smooth surface in the regenerated tendon; (2) presence of the well-repaired collagen bundle in regenerated tendon, but incontinuity in epidermis disturbed by adhesion tissues; (3) presence of unaligned tendon collagen fiber bundle and partial broken structure in the tendon; and (4) failed healing tendon. All observations and scoring processes were performed by three investigators independently.

Hematoxylin-eosin (HE) staining and Masson’s trichrome staining were performed through routine processes. For immunohistochemical staining, tissue sections were deparaffinized and rehydrated through standard procedures and successively antigen retrieved and blocked. Then, the sections were incubated with antibodies against α-smooth muscle actin (dilution: 1:100, Abcam, Cambridge, MA, USA) and collagen III (dilution: 1:100, Abcam, Cambridge, MA, USA) overnight at 4 °C. The next day, biotinylated secondary antibody (Servicebio, Wuhan, China) was applied to incubate the section for 1 h at room temperature. Color was developed using diaminobenzidine (Servicebio, Wuhan, China) before sections were counterstained with hematoxylin. Sections incubated with isotype control antibodies were used as the negative control. Six random sections from each group were selected for semiquantitative analysis. The percentage of positively stained cells for each group was determined using Image-Pro Plus 6.0 software (Media Cybernetics Corporation, USA) and then normalized by the percentage of positively stained cells in the adhesion group. The above-calculated fold change in each group was defined as the relative protein level.

### Biomechanical analysis of maximal tensile force

To evaluate the tendon healing quality, the maximal tensile force of the regenerated tendon was measured. Both proximal and distal ends of the Achilles tendon were attached to the clamps of the tensile testing system (Instron 5569, Norwood, MA, USA). A velocity of 10 mm/min was applied by the actuator to elongate the tendon. The amount of force to tendon rupture was recorded as the maximal tensile force. Five samples from each group were applied for analysis.

### Quantitative real-time polymerase chain reaction (qRT-PCR)

To isolate the total RNA from tissues or cells, TRIzol reagent (Invitrogen, Carlsbad, CA) was applied according to the manufacturer’s instructions. M-MLV reverse transcriptase (Takara Bio, Kusatsu, Japan) was used to accomplish the reverse transcription of 1 μg of total RNA. SYBR Green Premix Ex Taq (Takara Bio, Kusatsu, Japan) was then applied for target gene expression quantification. The gene expression fold change of each sample was referred to as relative gene expression and calculated using the 2^−ΔΔCT^ method. The experiment for each sample was run in triplicate. The primers used in this study were as follows, with GAPDH used as a housekeeping gene:

α-SMA: (Forward 5′-CACCATCGGGAATGAACGCTTC-3′, Reverse 5′-CTGTCAGCAATGCCTGGGTA-3′)

COL III: (Forward 5′-AGGTGGGTACACTGTAGCCT-3′, Reverse 5′-GATCGCATAGGTGACAGGTGTT-3′)

GAPDH: (Forward 5′-CACTGAGCATCTCCCTCACAA-3′, Reverse 5′-TGGTATTCGAGAGAAGGGAGG-3′)

### Western blotting analysis

WB analysis was performed as previously described [25]. Briefly, to isolate proteins from tissues, cells, or EVs, RIPA lysis buffer (Beyotime, Shanghai, China) was added to a cocktail of proteinase inhibitors (Yeasen, Shanghai, China), and the isolation process was conducted on ice. Proteins were acquired by centrifuging the lysate and harvesting the supernatants. The protein concentrations in the supernatants were detected by BCA assay. Twenty micrograms of total protein in each sample was subjected to sodium dodecyl sulfate-polyacrylamide gel electrophoresis (SDS-PAGE). The proteins in gels were electrotransferred onto polyvinylidene fluoride (PVDF) membranes. Then, 5% nonfat milk was used to block the PVDF membranes for 1 h, and the membranes were incubated with primary antibodies against Col III (1:1000), α-SMA (1:1000), CD9 (1:1000), CD63 (1:1000), ALIX (1:1000), TSG101 (1:1000), calnexin (1:1000), GRP78 (1:1000), CHOP (1:1000), BAX (1:1000), and Bcl-2 (1:1000) overnight at 4 °C. Horseradish peroxidase (HRP)-conjugated secondary antibodies (Servicebio, Wuhan, China, dilution: 1:3000) were then applied to incubate with the membranes for 1 h at ambient temperature. Chemiluminescent signals were developed with the enhanced chemiluminescence reagent (Epizyme, Shanghai, China) before being captured by the ChemiDoc CRS imaging system (Bio-Rad, USA). Experiments were performed independently three times. For semi-quantification of the WB results, the relative protein expression was calculated in the form of relative gray level using ImageJ software (Version 1.32, National Institutes of Health). Briefly, the images were initially prepared with the “subtract background” and “invert” functions in ImageJ. Then, each protein band of interest was chosen, and integrated optical density (IntDen) was “measured”. The relative gray level was defined as the normalized value of the IntDen of interest protein to the IntDen of the internal control protein β-actin.

### Statistical analysis

GraphPad Prism 7 was used to perform the statistical analysis. Data are shown as the means ± standard deviation. Multiple comparisons were achieved using one-way ANOVA, and Tukey’s test was chosen for post hoc comparisons. Comparison of two groups was finished using Student’s *t* test. Statistical significance was set at *P* value < 0.05. All experiments were independently performed at least three times.

## Results

### Identification and characterization of the isolated EVs

**Fig. 1:**
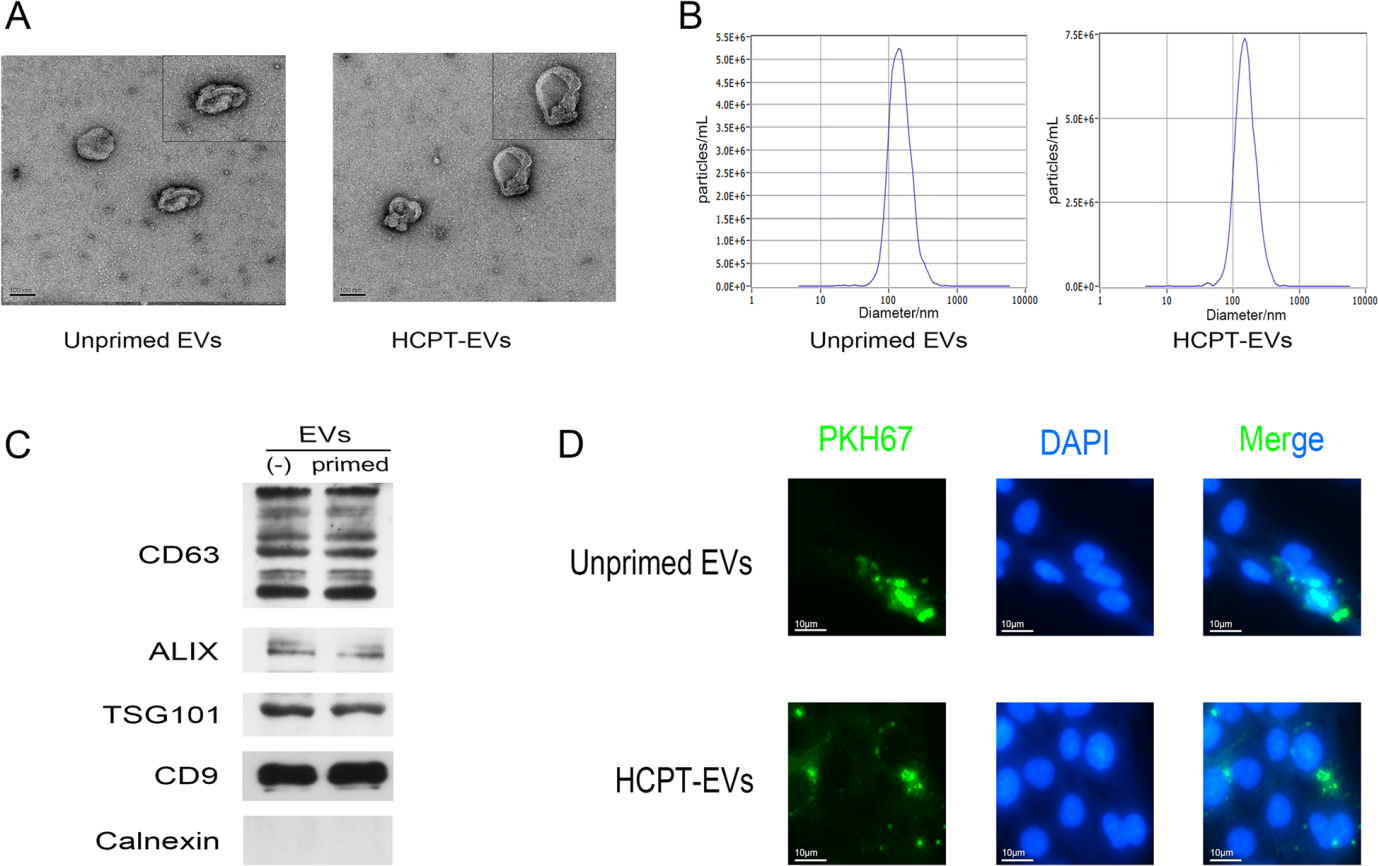
Identification and characterization of extracellular vesicles (EVs). **a** Representative electron microscopic image of isolated EVs. **b** NTA analysis showing the size distribution of EVs. **c** Western blotting (WB) analysis of extracellular markers, including CD9, CD63, ALIX, calnexin, and TSG101. **d** PKH67 staining showing the cellular uptake of EVs

Typical cup-shaped double membrane-bound structures of EVs were observed in both Unprimed EVs and HCPT-EVs with no evident differences in morphology as revealed by TEM (Fig. 1a). The majority particle size of both Unprimed EVs and HCPT-EVs was determined to be between 30 and 150 nm by NTA, which also showed no prominent differences between Unprimed EVs and HCPT-EVs (Fig. 1b). Protein expression of EV markers including CD9, CD63, ALIX, and TSG101 was abundant, while the endoplasmic reticulum marker calnexin was absent in either Unprimed EVs or HCPT-EVs, all confirming the satisfactory isolation of EVs (Fig. 1c). Cellular uptake experiments by PKH67 staining of EVs further indicated that both Unprimed EVs and HCPT-EVs could be successfully internalized by fibroblasts (Fig. 1d).

### HCPT-EVs demonstrated promoted beneficial effects in a rat model of tendon injury

**Fig. 2:**
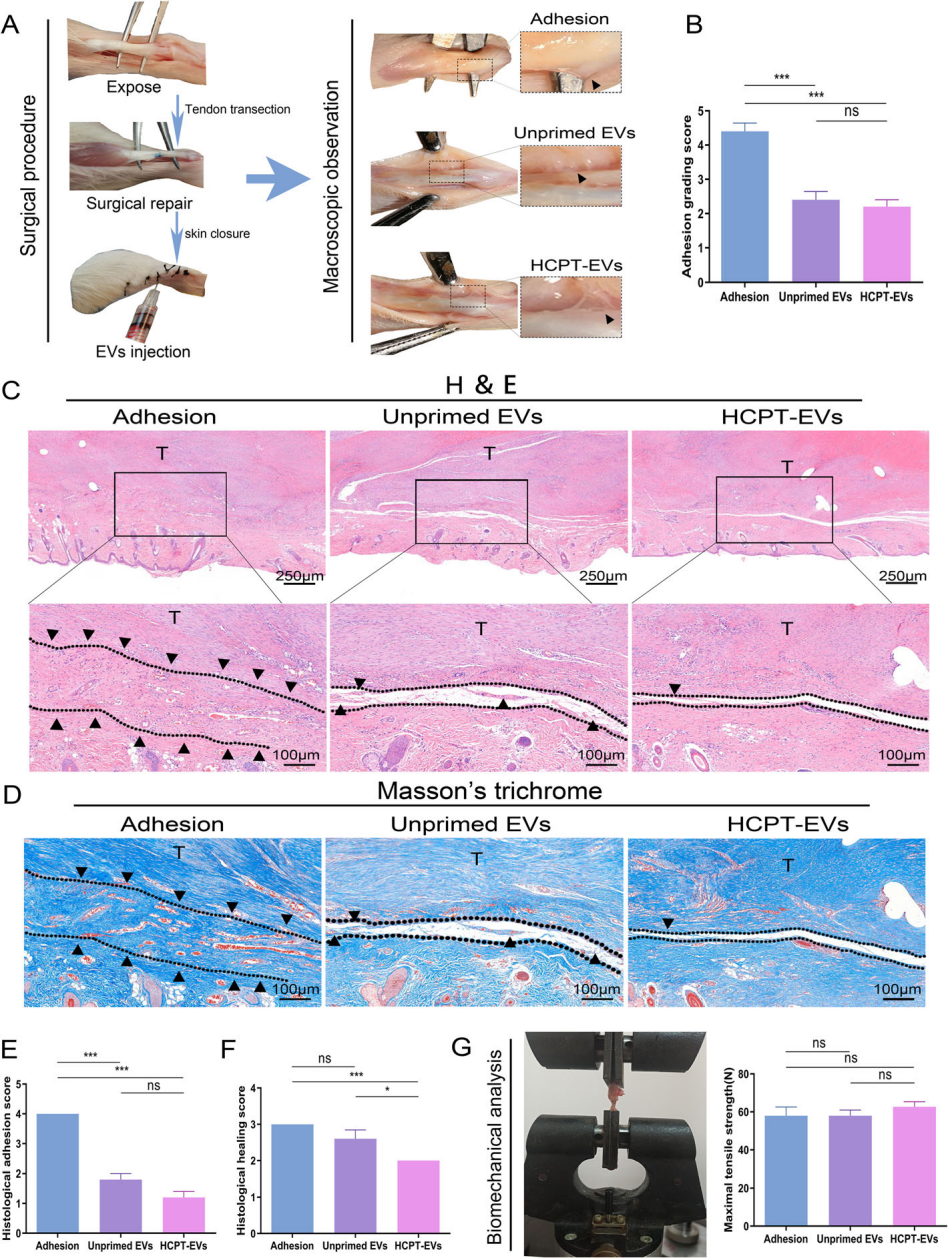
EVs derived from hydroxycamptothecin-primed human umbilical cord stem cells (HCPT-EVs) more effectively ameliorated tendon adhesion in a rat model of tendon injury. EVs derived from unprimed human umbilical cord mesenchymal stem cells (Unprimed EVs), HCPT-EVs, or PBS were subcutaneously injected into the injury site, and tendon tissues were harvested at 3 weeks postsurgery. **a** Representative images showing surgical procedures and macroscopic observation of the repaired tendon and concomitant adhesion. **b** Adhesion grading score (*n* = 5 per group) for macroscopic rating of tendon adhesion. **c** Hematoxylin-eosin (HE) staining for tendon adhesion observation. Lower panels are magnified images of the boxed areas indicated in the upper panel. **d** Masson’s trichrome staining for tendon adhesion observation. Histological rating of tendon adhesion and healing using, **e** histological adhesion score (*n* = 5 per group), **f** histological healing score (*n* = 5 per group), and **g** biomechanical testing for maximal tensile strength of repaired tendon (*n* = 5 per group). The capital letter “T” denotes tendon. The dotted lines represent the boundaries between the tendon and peritendinous tissues and encapsulate the adhesion tissues. Black triangles indicate the adhesive tissues. *P < 0.05, ***P < 0.001; ns, not significant

The anti-adhesion effect of HCPT-EVs was validated in a rat Achilles tendon injury model (Fig. 2a). As expected, macroscopic observation showed that both HCPT-EVs and Unprimed EVs effectively attenuated tendon adhesion to peritendinous tissues (Fig. 2a, b). Further HE and Masson’s trichrome staining of the histological sections revealed that both the histological boundaries between the Achilles tendon and the peritendinous tissues were more distinct and that the regenerated tendon was more continuous and less variable in the HCPT-EVs group than in the Unprimed EVs group (Fig. 2c, d). Rating of the adhesion degree was performed using histological adhesion scores based on histological findings. The results showed that both Unprimed EVs treatment and HCPT-EVs treatment dramatically lowered the adhesion grade of the tendon. Comparing the scores achieved by HCPT-EVs with Unprimed EVs showed a tendency toward decreasing, although it was not significant (Fig. 2e). Surprisingly, HCPT-EVs treatment resulted in a significantly lower histological healing score than Unprimed EVs treatment (Fig. 2f), although the maximal tensile strength of the regenerated tendon remained the same among the three groups (Fig. 2g).

### HCPT-EVs more potently restored the normal fibroblast growth rate upon TGFβ stimulation

**Fig. 3:**
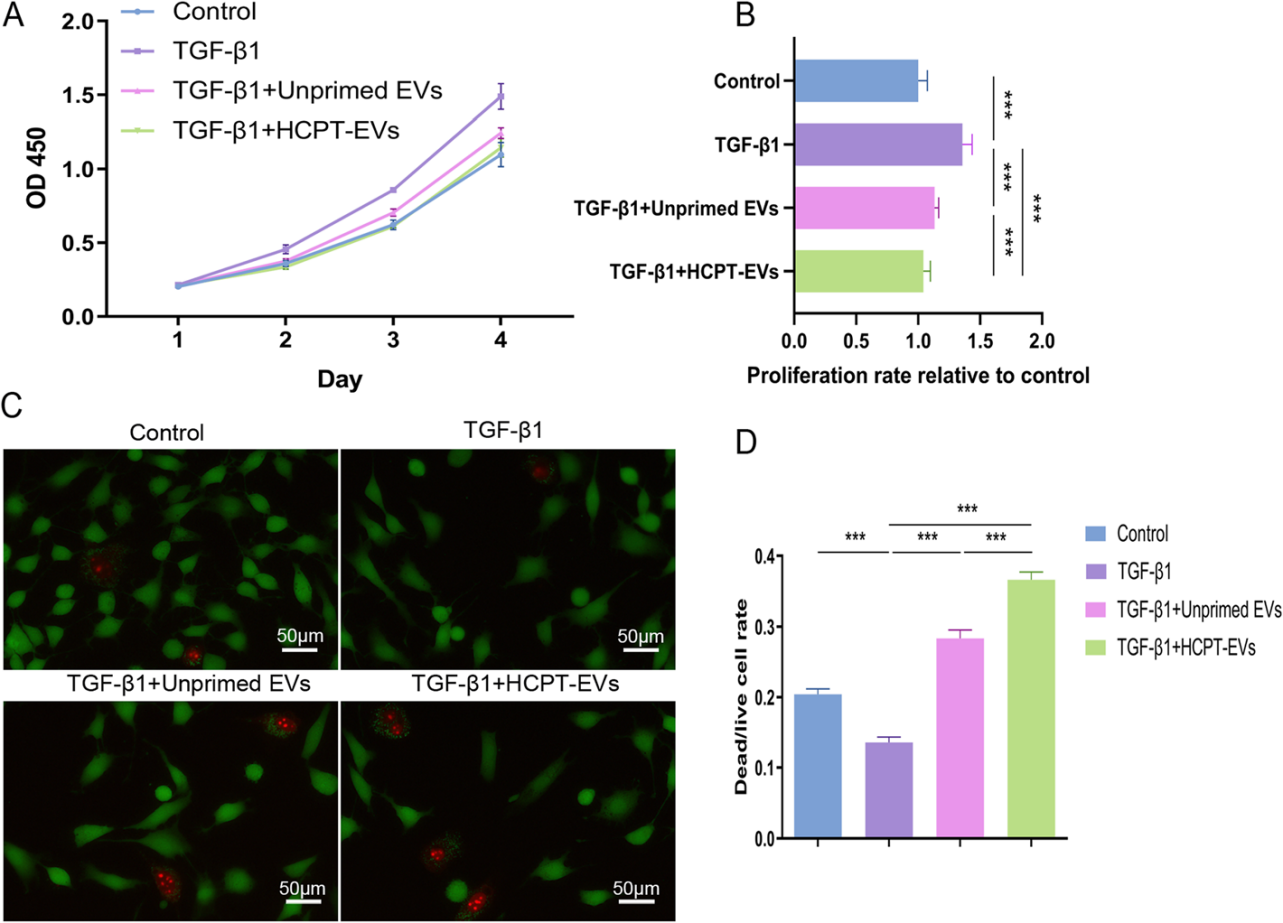
HCPT-EVs are more capable of repressing fibroblast proliferation and viability boosted by TGF-β1. Rat fibroblasts were treated with Unprimed EVs or HCPT-EVs (100 μg/mL) in the presence of TGF-β1 (2 ng/ml) for the indicated times and for 48 h in a live/dead assay. **a** Cell proliferation curve of fibroblasts plotted according to CCK-8. **b** Proliferation rate relative to the control. **c** Representative dead/live staining of fibroblasts. **d** Dead/live rate of fibroblasts. *P < 0.05, ***P < 0.001; ns, not significant

Fibroblast cell proliferation and viability were checked in vitro as possible fibrotic processes affected by HCPT-EVs. The results shown by CCK-8 indicated that increased cell proliferation induced by TGF-β1 was significantly reduced by either Unprimed EVs or HCPT-EVs supplementation. It should also be noted that HCPT-EVs have a stronger efficacy than Unprimed EVs and almost eliminate the promoting effect of TGF-β1 on fibroblast proliferation (Fig. 3a, b). Fibroblast cell viability evaluation was accomplished by live/dead staining. The results also suggested that both Unprimed EVs and HCPT-EVs damaged the cell viability enhanced by TGF-β1. Similarly, HCPT-EVs supplementation resulted in a greater dead/live rate in fibroblasts than Unprimed EVs (Fig. 3c, d).

### HCPT-EVs more effectively decreased the myofibroblast activation induced by TGFβ after tendon injury

**Fig. 4:**
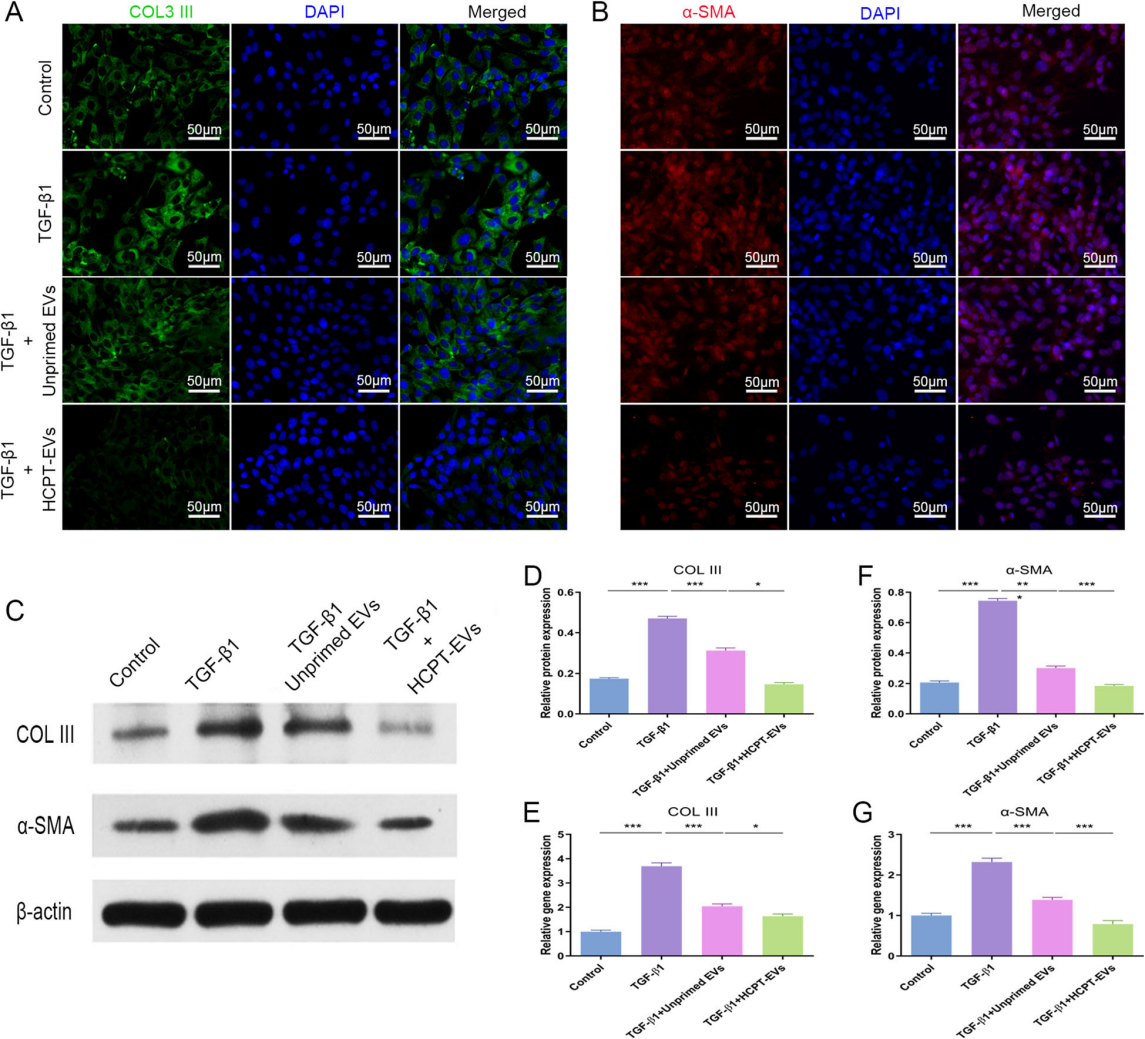
HCPT-EVs showed an enhanced inhibitory effect on myofibroblast activation induced by TGF-β1. Rat fibroblasts were incubated with Unprimed EVs or HCPT-EVs (100 μg/mL) in the presence of TGF-β1 (2 ng/ml) for 24 or 48 h. **a**, **b** Immunofluorescent staining of COL III and α-SMA. **c** WB analysis of COL III and α-SMA. **d**, **f** Semiquantitative analysis of WB results. **e**, **g** qRT-PCR analysis of COL III and α-SMA expression. *P < 0.05, **P < 0.01, ***P < 0.001; ns, not significant

To explore whether HCPT-EVs could more effectively influence myofibroblast activation and the subsequent collagen deposition process after tendon injury, the expression of COL III and α-SMA was first checked in vitro. Upon TGF-β1 stimulation, both COL III and α-SMA were conspicuously induced in fibroblasts and reflected the transformation of fibroblasts to myoblasts. However, the addition of Unprimed EVs or HCPT-EVs markedly reversed the above profibrotic changes, causing weaker staining of both COL III and α-SMA (Fig. 4a, b). This inhibitory phenomenon was more obvious after the addition of HCPT-EVs than Unprimed EVs, which was verified by WB analysis (Fig. 4c, d, f). qRT-PCR analysis further suggested that Unprimed EVs and HCPT-EVs suppressed COL III and α-SMA at the transcription level and displayed a larger decreasing trend after HCPT-EVs administration than Unprimed EVs (Fig. 4e, g).

**Fig. 5:**
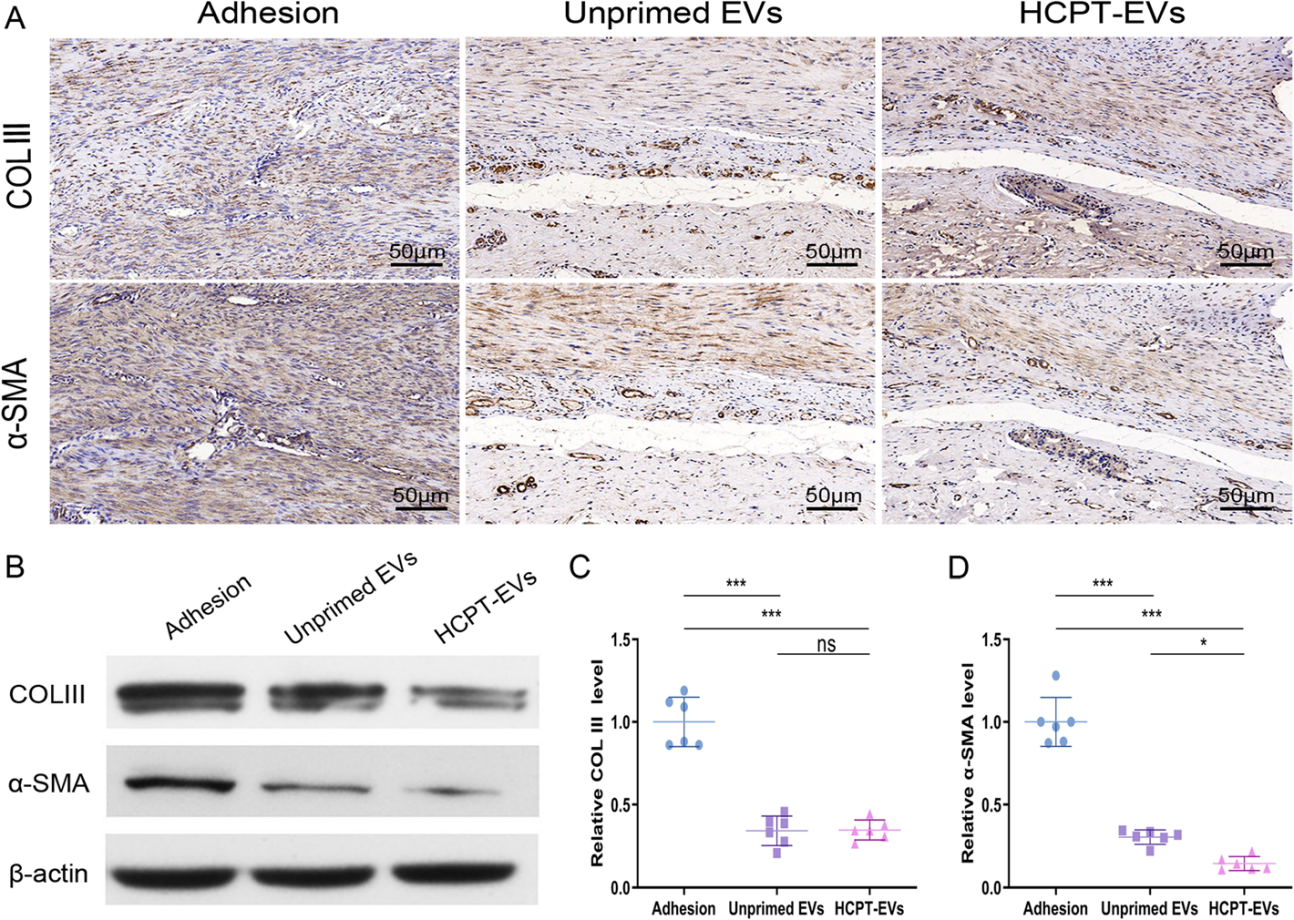
HCPT-EVs obtained superior capacity to protect tendons from myoblast activation after tendon injury. The tendon injury sites were treated topically with Unprimed EVs, HCPT-EVs, or PBS, and tendon tissues were collected 3 weeks after surgery. **a** Immunohistochemical staining of COL III and α-SMA in peritendinous tissues. **b** WB analysis of COL III and α-SMA in peritendinous tissues. **c**, **d** Semiquantitative analysis for immunohistochemical staining of COL III and α-SMA expression (*n* = 6 per group). *P < 0.05, ***P < 0.001; ns, not significant

The effect of Unprimed EVs or HCPT-EVs on collagen deposition was then evaluated again in the rat model of Achilles tendon injury. In accordance with the in vitro study, tendon adhesion following tendon injury was accompanied by massive expression of COL III and α-SMA, which was significantly diminished by Unprimed EVs or HCPT-EVs treatment as proposed by both immunohistochemical staining and WB analysis (Fig. 5a, b). The semiquantitative results of immunohistochemical staining also demonstrated that HCPT-EVs treatment gained less expression of α-SMA than Unprimed EVs treatment, showing that HCPT-EVs had a better inhibitory effect on myofibroblast transformation (Fig. 5c, d).

### Activation of the ERS pathway partially contributes to the anti-adhesion effect of HCPT-EVs

**Fig. 6:**
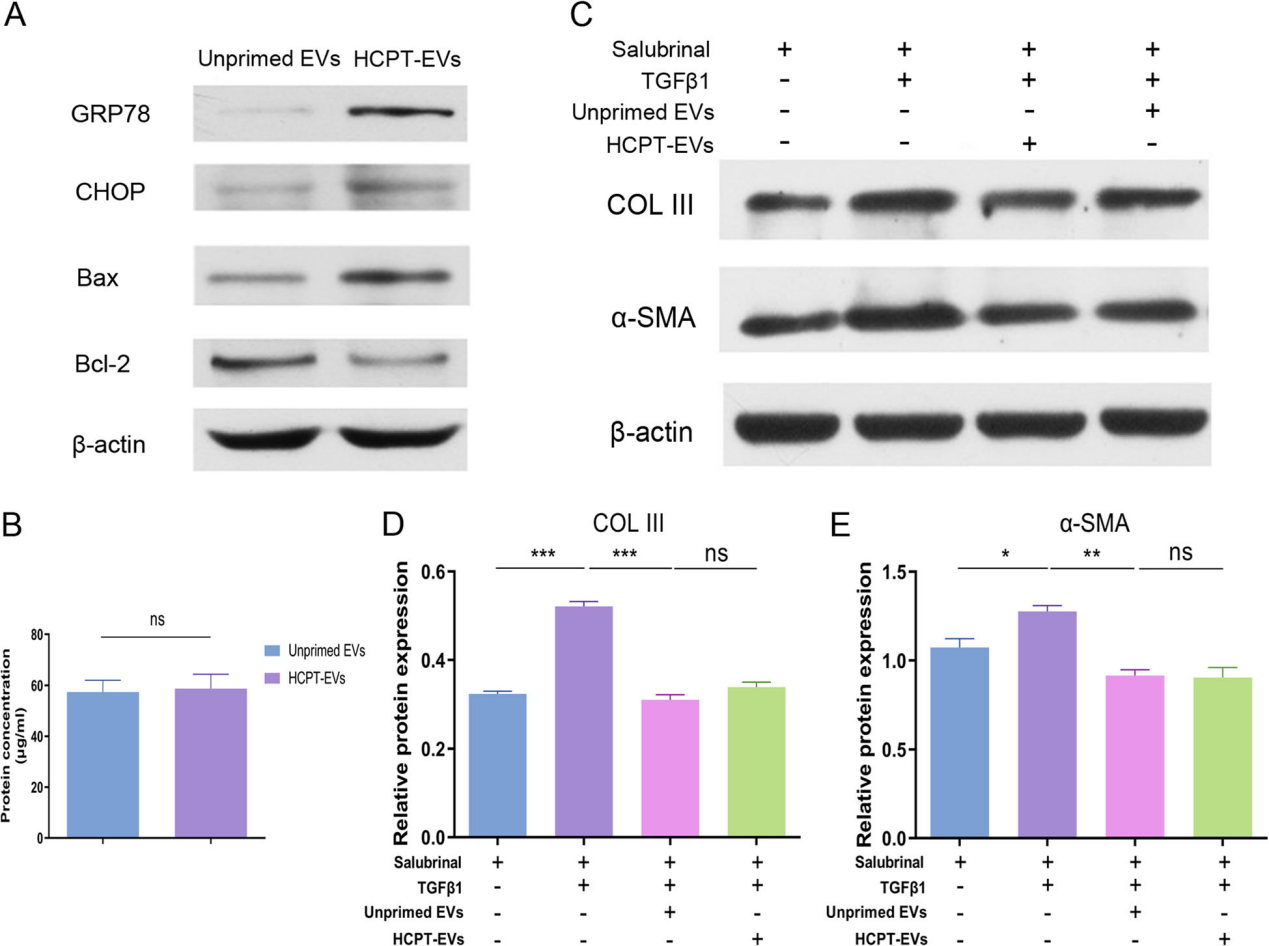
The improved anti-adhesion effect of HCPT-EVs was attributed to activation of the ERS pathway. **a** Representative WB images of GRP78, CHOP, Bax, and Bcl-2. **b** BCA was applied to determine the total protein concentration of EVs. Rat fibroblasts were treated with salubrinal (10 μM) for 1 h before incubation with Unprimed EVs or HCPT-EVs (100 μg/mL) in the presence of TGF-β1 (2 ng/ml) for 48 h. **c** Representative WB images of COL III and α-SMA. **d**, **e** Semiquantitative analysis of COL III and α-SMA. *P < 0.05, **P < 0.01, ***P < 0.001; ns, not significant

To answer why the HCPT-EVs displayed better anti-adhesion effects than Unprimed EVs, we further compared the changes in protein concentrations and compositions of EVs from HUMSCs with or without HCPT pretreatment. However, the total protein concentrations were found to be at the same level for the Unprimed EVs and HCPT-EVs (Fig. 6b), suggesting that a compositional change in EVs contents might be the key reason. Protein detection by WB analysis then demonstrated that the ERS pathway effector protein GRP78 and CHOP along with the pro-apoptotic protein Bax were evidently upregulated, while the anti-apoptotic protein Bcl-2 was downregulated in the HCPT-EVs compared with the Unprimed EVs (Fig. 6a), indicating that an ERS pathway-dependent mechanism might be involved in the enhanced anti-adhesion effect of HCPT-EVs. To test this hypothesis, a selective eIF2α dephosphorylation inhibitor, salubrinal, was used to inactivate the ERS pathway in fibroblasts. WB analysis proposed that although the inhibitory effect of HCPT-EVs on TGF-β1-induced COL III and α-SMA expression remained and showed significance, the superior performance of HCPT-EVs over Unprimed EVs disappeared in the presence of salubrinal (Fig. 6c–e). Therefore, we can infer that the anti-adhesion effect of HCPT-EVs was only partially ERS pathway-dependent and that the activation of the ERS pathway could account for the preferable anti-adhesion effect of HCPT-EVs to Unprimed EVs.

## Discussion

Currently, with the booming economy and thriving traffic, there is an increasing rate of limb trauma. Unfortunately, tendon injury is difficult to fully recover from and is often accompanied by a fibrotic healing process characterized by the initial inflammatory responses and subsequent myofibroblast activation and collagen deposition, causing tendon adhesion with the peritendinous tissues [26, 27], which, given the lack of preventive methods, can require a second myotenolysis surgery. As ideal effector molecule carriers and biocompatible therapeutic tools with pure biogenic derivation, EVs are becoming rising stars in treating illness, showing pleiotropic function and prompting encouraging results in various organs and systems [28–30]. Tendon repair has already been found to be promoted by EVs from BMSCs and ADSCs [9, 10, 31]. Motivated by the powerful repair and mass production capabilities of HUMSCs, HUMSC EVs have also been widely studied and applied in regenerative medicine [32, 33]. Recently, the anti-fibrosis ability of HUMSC EVs has also been discovered. Treatment with HUMSC EVs effectively suppressed fibrotic responses in the kidney and liver, thereby facilitating their recovery from acute injury [25, 34]. Nonetheless, to date, few reports have investigated the effect of HUMSC EVs on tendon adhesion. Here, in this study, we proved that HUMSC EVs attenuated tendon adhesion in a rat model of Achilles tendon injury, as revealed by the reappearance of clear boundaries between tendon and peritendinous tissues according to histological observation.

Among the strategies known to modify bioactive cargos and augment the treatment efficacy of EVs, including electroporation and genetic manipulation [35, 36], cell pretreatment is easy and economical to perform with high efficiency. Wang Z et al. demonstrated that cyclic stretch force primed periodontal ligament cells can secrete improved EVs to inhibit proinflammatory NF-κB signaling and IL-1β production [37]. Simple atorvastatin preincubation strengthened the angiogenesis-promoting potential of EVs from mesenchymal stem cells in acute myocardial infarction [38]. Interferon-gamma (IFN-γ) priming is also reported to potentiate the anti-inflammatory and pro-healing effects of EVs from ADSCs [31]. Since HCPT was identified as a potent tendon adhesion inhibitor in our recent study [6], we suggested that pretreatment of HUMSCs with HCPT could further enhance the anti-adhesion efficacy of their secreted EVs, namely, HCPT-EVs. As proof of our concept, the application of HCPT-EVs overall manifested superior adhesion inhibitory effects to Unprimed EVs. A stronger suppressive effect on the viability and overproliferation of fibroblasts induced by profibrotic TGF-β signaling was obtained by HCPT-EVs, benefiting preferable separation from the invading peritendious tissues in HCPT-EVs treated tendons. Meanwhile, the regenerated tendons administered HCPT-EVs were more organized, earning lower histological healing scores than Unprimed EVs. The improved healing ability of tendons may be explained by the optimized balancing of intrinsic and extrinsic tendon healing after HCPT-EVs delivery, where successful control of fibrotic tendon healing (extrinsic healing) made way for natural tenocyte proliferation and regeneration (intrinsic healing).

Numerous studies have established that myofibroblasts are the major effector cells in tissue adhesion; they are α-SMA-positive and feature increased collagen secretion, contributing to overwhelming extracellular matrix deposition during tendon healing [26, 39, 40]. TGF-β is suggested to be the primary pathological mediator that promotes fibroblast viability maintenance, proliferation, and fibroblast-to-myofibroblast transformation [40, 41]. Using TGF-β1 as a stimulant, we mimicked the fibroblast activation state in vitro and found that HCPT-EVs abated the rapid proliferation of fibroblasts promoted by TGF-β1 and damaged the viability-maintaining ability of TGF-β1 to a larger extent than Unprimed EVs. The myofibroblast transformation induced by TGF-β1 was also hampered by HCPT-EVs. In accordance with the in vitro results, impaired myofibroblast transformation and collagen deposition were also observed in vivo, as revealed by the decreased COL III and α-SMA expression, with HCPT-EVs exerting a greater effect than Unprimed EVs.

Conditional pretreatment of producer cells can either change the total protein yields or the components encapsulated in the secreted vesicles. It has been reported that hypoxia pretreatment increased the productivity of EVs from renal tubular epithelial cells but failed to produce the same effect in cardiovascular cells [42, 43]. However, thrombin instead of hypoxia optimally promotes EVs’ generation by mesenchymal stem cells [44]. Hence, the rationale behind modified EVs’ functionality differs and hinges on different treatment schemes and producer cell types and origins. In our study, we showed that pretreatment of HUMSCs with HCPT did not increase the total protein concentrations of their secreted EVs, indicating a possible change in vesicle content composition. Given the previously recognized involvement of ERS signaling in the mechanisms of HCPT functioning [6], we questioned whether HCPT-EVs incorporate ERS signaling effector proteins. ERS typically occurs when misfolded or unfolded proteins accumulate in the endoplasmic reticulum and prompt the master coordination adaptive program called the unfolded protein response (UPR), which activates ERS signaling to process these unwanted proteins to restore homeostasis [45]. However, exceeding the load of ERS can fall beyond the tolerance of the UPR and directly give rise to pro-apoptosis signaling [45, 46]. During this process, glucose-regulated protein 78 (GRP78/BiP) stands as the ER chaperone protein that increases in response to ERS and is thus a sensitive indicator of ERS signaling activation [47]. C/EBP homologous protein (CHOP/GADD153) is located downstream of ERS signaling and can control the pro-apoptotic Bax/Bcl-2 ratio [46]. Coherently, comparing the protein composition between Unprimed EVs and HCPT-EVs revealed dramatically more encapsulation of GRP78 and CHOP in HCPT-EVs than in Unprimed unprimed EVs. Consistent with this, the Bax/Bcl-2 ratio in vesicles was also elevated, which might portend the reinforced pro-apoptosis ability of HCPT-EVs. To further confirm that the increased load of ERS signaling effector proteins in HCPT-EVs was responsible for their action on fibroblasts, the ERS antagonist salubrinal was applied and reversed the inhibitory effect of HCPT-EVs on COL III and α-SMA expression in fibroblasts, suggesting the restoration of the myofibroblast phenotype. These results revealed that the anti-adhesion effect of HCPT-EVs may also be ERS signaling relevant and dependent, similar to that of HCPT.

Some limitations must be stated in our study. Most importantly, the identified ERS signaling-dependent anti-adhesion mechanism of HCPT-EVs was not corroborated in vivo, and the precise regulatory process needs to be further dissected. Then, the treatment period and doses of HCPT-EVs should be optimized in subsequent studies. In addition, the proposed ERS signaling-dependent mechanism was enlightened by our previous findings on HCPT, and further high-throughput sequencing or mass spectrum analysis of the EVs may add more value to understanding the mechanisms of HCPT-EVs wielded in the prevention of tendon adhesion. Finally, the potential involvement of HCPT in the content and effects of EVs cannot be fully excluded, although some studies have suggested that the loading of small molecular drugs to EVs by simple cell and drug coculture is of low efficiency without electroporation or squeezing.

## Conclusion

**Fig. 7:**
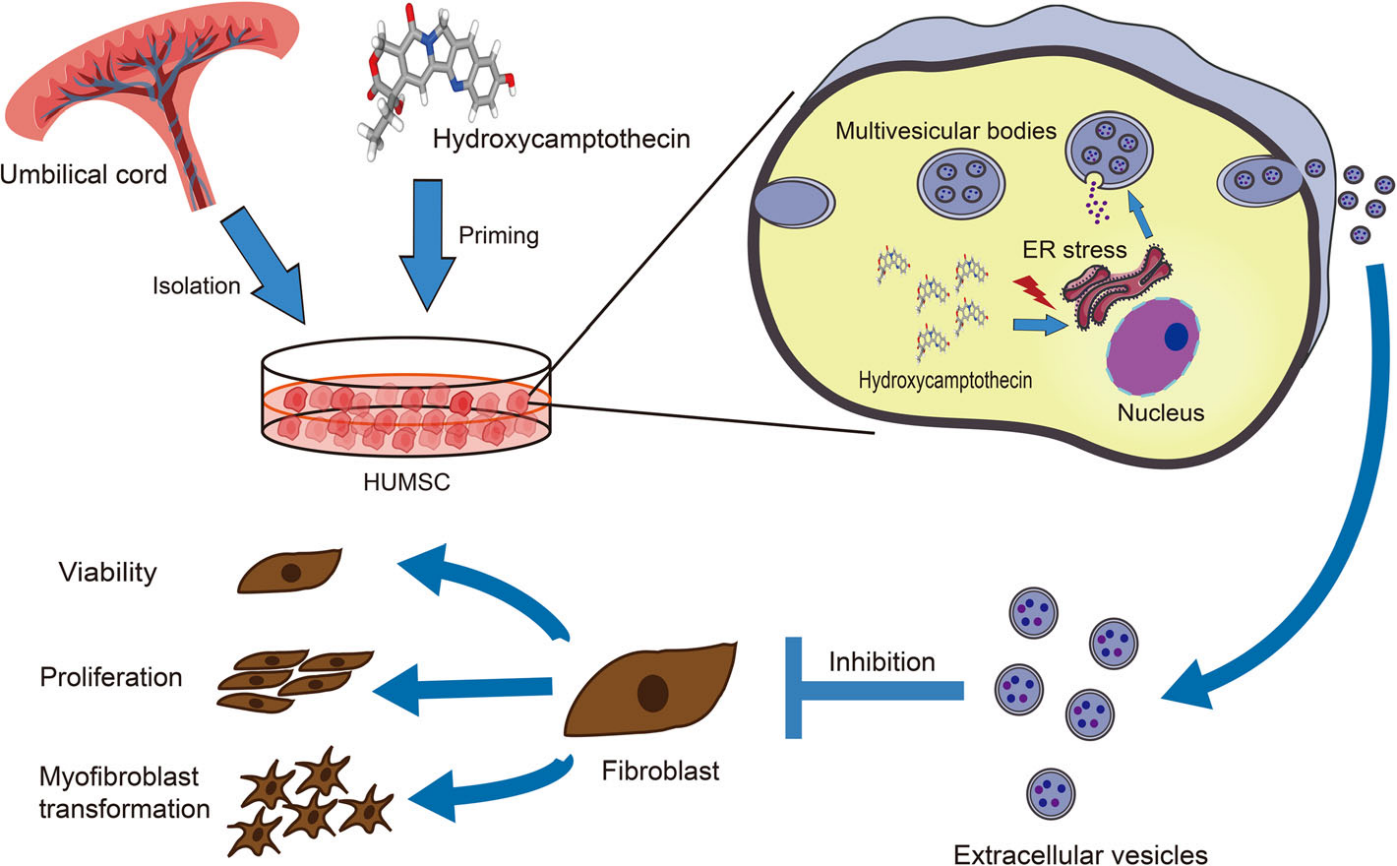
Scheme of the production of HCPT-EVs that prevents tendon adhesion by inhibiting fibroblast viability, proliferation, and myofibroblast transformation. After hydroxycamptothecin priming of human umbilical mesenchymal stem cells (HUMSCs), endoplasmic reticulum (ER) stress was elicited and led to more encapsulation of ER stress effector protein into the secreted EVs, which enhanced the anti-viability, anti-proliferation, and myofibroblast transformation inhibitory potential of EVs

The findings in our study demonstrated that HCPT-EVs possessed superior anti-adhesion capability relative to Unprimed EVs in a rat model of tendon injury. In vitro analysis proved that HCPT-EVs could more effectively inhibit fibroblast proliferation, viability, and collagen deposition upon TGF-β1 stimulation compared with Unprimed EVs, which is dependent on the ERS pathway as utilized by HCPT (Fig. 7). Considering their high biocompatibility and easy use, HCPT-EVs represent an intriguing option for anti-adhesion therapy.

## Abbreviations

HCPT: Hydroxycamptothecin
HUMSC: Human umbilical cord mesenchymal stem cell
ADSCs: Adipose tissue-derived mesenchymal stem cells
BMSCs: Bone marrow mesenchymal stem cells
EVs: Extracellular vesicles
ERS: Endoplasmic reticulum stress
NTA: Nanoparticle tracking analysis
TEM: Transmission electron microscope
α-SMA: α-Smooth muscle actin
GRP78: Glucose-regulated protein 78
PBS: Phosphate-buffered saline
WB: Western blotting
qRT-PCR: Quantitative real-time PCR
DAPI: 4′,6-Diamidino-2-phenylindole
HE: Hematoxylin-eosin
